# Supplementary material for: BNT162b2 mRNA COVID-19 Vaccine Effectiveness in Patients with Coeliac Disease Autoimmunity: Real-World Data from Mass Vaccination Campaign
Source: Viruses. 2023 Sep 21;15(9):1968. doi: 10.3390/v15091968 (PMC10534420; doi:10.3390/v15091968)
Supplement: Supplementary file 1 [file viruses-15-01968-s001.zip › viruses-2589001-supplementary.pdf]

viruses

MDPI

Table S1. Cox regression analysis of patients with likely well-controlled coeliac disease (n = 3190) and their matched patients (n = 8, 923).

	***P*-value**	**Hazard Ratio**	**95% Confidence Interval**	
			Lower	Upper
Likely well-controlled coeliac patients	0.34	0.90	0.71	1.13
BMI normal	Reference			
BMI underweight	0.45	1.14	0.81	1.6
BMI over	0.11	0.81	0.63	1.05
BMI obese	0.08	0.74	0.52	1.04
BMI missing	0.79	0.94	0.59	1.49
Diabetes	0.36	0.74	0.39	1.42
Hypertension	0.05	0.59	0.35	0.99
Cancer	0.26	0.67	0.34	1.33
Cardiovascular disease	0.45	0.73	0.32	1.66
Chronic Kidney Disease	0.76	0.91	0.48	1.7
Immunocompromised	0.06	0.42	0.17	1.05

Table S2. Cox regression analysis for patients with near-certain coeliac disease (n = 2310) and their matched patients (n = 6423).

	***p*-value**	**Hazard Ratio**	**95% Confidence Interval**	
			Lower	Upper
Near-certain Coeliac disease	0.13	0.81	0.62	1.07
BMI normal	Reference			
BMI underweight	0.27	1.25	0.84	1.90
BMI over	0.42	0.89	0.67	1.19
BMI obese	0.08	0.69	0.46	1.04
BMI missing	0.14	0.62	0.33	1.18
Diabetes	0.67	1.14	0.59	2.21
Hypertension	0.03	0.51	0.28	0.93
Cancer	0.28	0.63	0.27	1.46
Cardiovascular disease	0.74	0.86	0.34	2.14
Chronic Kidney Disease	0.37	0.69	0.32	1.54
Immunocompromised	0.16	0.43	0.13	1.40

Table S3. Cox regression analysis for patients with likely not well-controlled coeliac disease (n = 162) and their matched patients (n = 458).

	***P*-value**	**Hazard Ratio**	**95% Confidence Interval**	
			Lower	Upper
Likely not well-controlled coeliac patients	0.32	0.57	0.19	1.71
BMI normal	0.20			
BMI underweight	0.02	5.17	1.36	19.64
BMI over	0.56	1.37	0.48	3.87
BMI obese	0.52	1.47	0.46	4.72
BMI missing	0.96	1.05	0.13	8.61
Diabetes	0.66	0.62	0.08	5.14

Hypertension	0.75	0.79	0.18	3.44
Cancer	0.60	0.54	0.05	5.34
Cardiovascular disease	0.90	1.16	0.12	11.14
Chronic Kidney Disease	0.62	1.56	0.26	9.11
Immunocompromised	0.30	2.48	0.43	14.06

Table S4. Cox regression analysis for patients with newly diagnosed coeliac disease (n = 162) and their matched patients (n = 458).

	***P*-value**	**Hazard Ratio**	**95% Confidence Interval**	
			Lower	Upper
Likely newly diagnosed coeliac patients	0.98	0.99	.51	1.93
BMI normal	0.63			
BMI underweight	0.76	0.80	0.19	3.40
BMI over	0.35	1.36	0.71	2.62
BMI obese	0.42	0.61	0.18	2.04
BMI missing	0.57	0.66	0.15	2.81
Diabetes	0.98	0.00	0.00	
Hypertension	0.27	0.32	0.04	2.41
Cancer	0.98	0.00	0.00	.
Cardiovascular disease	0.12	3.20	0.74	13.90
Chronic Kidney Disease	0.98	0.00	0.00	.
Immunocompromised	0.52	1.94	0.26	14.23
